# Intensity of space use reveals conditional sex‐specific effects of prey and conspecific density on home range size

**DOI:** 10.1002/ece3.2032

**Published:** 2016-03-28

**Authors:** Malin Aronsson, Matthew Low, José V. López‐Bao, Jens Persson, John Odden, John D. C. Linnell, Henrik Andrén

**Affiliations:** ^1^ Department of EcologySwedish University of Agricultural SciencesGrimsö Wildlife Research StationSE‐73091RiddarhyttanSweden; ^2^ Department of EcologySwedish University of Agricultural SciencesSE‐75007UppsalaSweden; ^3^ Research Unit of Biodiversity (UO/CSIC/PA)Oviedo UniversityMieres33600Spain; ^4^ Norwegian Institute for Natural ResearchSluppenNO‐7585TrondheimNorway

**Keywords:** *Capreolus capreolus*, carnivore, kernel home range estimator, ranging behavior, spatiotemporal variation, utilization distribution

## Abstract

Home range (HR) size variation is often linked to resource abundance, with sex differences expected to relate to sex‐specific fitness consequences. However, studies generally fail to disentangle the effects of the two main drivers of HR size variation, food and conspecific density, and rarely consider how their relative influence change over spatiotemporal scales. We used location data from 77 Eurasian lynx (*Lynx lynx*) from a 16‐year Scandinavian study to examine HR sizes variation relative to prey and conspecific density at different spatiotemporal scales. By varying the isopleth parameter (intensity of use) defining the HR, we show that sex‐specific effects were conditional on the spatial scale considered. Males had larger HRs than females in all seasons. Females' total HR size declined as prey and conspecific density increased, whereas males' total HR was only affected by conspecific density. However, as the intensity of use within the HR increased (from 90% to 50% isopleth), the relationship between prey density and area showed opposing patterns for females and males; for females, the prey density effect was reduced, while for males, prey became increasingly important. Thus, prey influenced the size of key regions within male HRs, despite total HR size being independent of prey density. Males reduced their HR size during the mating season, likely to remain close to individual females in estrous. Females reduced their HR size postreproduction probably because of movement constrains imposed by dependent young. Our findings highlight the importance of simultaneously considering resources and intraspecific interactions as HR size determinants. We show that sex‐specific demands influence the importance of prey and conspecific density on space use at different spatiotemporal scales. Thus, unless a gradient of space use intensity is examined, factors not related to total HR size might be disregarded despite their importance in determining size of key regions within the HR.

## Introduction

Access to critical resources is an essential determinant of individual fitness, with spacing behavior being a key factor regulating this access (Morales et al. 2010). Because of its central role in influencing population dynamics and distribution, home range (HR) size has been extensively studied. Differences in body size, diet, social organization, and mating system explain general HR size variation between species (McNab 1963; Clutton‐Brock and Harvey 1978; Kelt and Van Vuren 2001; Carbone et al. 2011), while resource distribution, abundance, and predictability together with density of competing conspecifics are important drivers of HR size variation within species (Maher and Lott 2000; McLoughlin and Ferguson 2000; Jetz et al. 2004; Mitchell and Powell 2004; López‐Bao et al. 2014).

**Figure 1:**
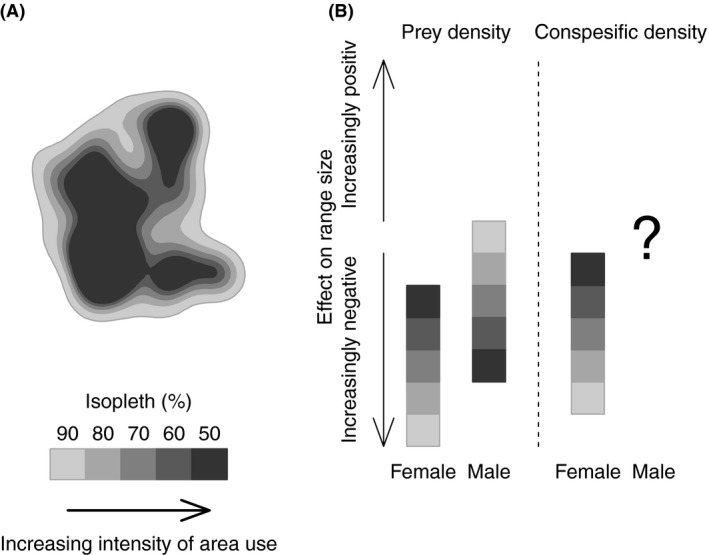
Home range (HR) estimation obtained as a probability density function of intensity of area use (A). We estimated lynx HR size at 5 use intensities represented by the 90% (total HR), 80%, 70%, 60%, and 50% isopleths where intensity of use increases with decreasing isopleth level (i.e., darker areas = higher use). We predict that range size determinants will vary within an animal's HR relative to the intensity of use (B). Female HRs should be just large enough to contain sufficient food resources to survive and nourish offspring, and hence, the negative effect of prey density on range size should be strongest on the total HR size (90% isopleth, light grey) and become less important with decreasing isopleth level. We predict the opposite pattern for males as total HR size is set to maximizing mating opportunities whereas basal energy needs should affect space use at a lower isopleth levels (darker grey). For females, we expect conspecific density to show its greatest effect on total HR size due to territorial behavior. For males, however, conspecific density represent both resources (females) and competitors (males), and thus, we expect the effect of conspecific density on male spacing behavior to vary with conspecific density *per se*.

Because resource distribution and conspecific interactions are not uniform, the relative importance of resources and conspecifics in relation to HR variation should change both in space and time (Börger et al. 2006a; van Beest et al. 2011; Campos et al. 2014). As HRs are often defined in terms of some minimum intensity of space use by the focal animal (Kie et al. 2010), critical insights can be gained by examining how the effect of range size determinants changes as intensity of space use changes within the HR (Fig. 1). For example, factors that are important for determining total HR may become less important in determining the size of more intensively used areas within the HR and vice versa (i.e., compared to the second‐ and third‐order habitat selection; Johnson 1980). Similarly, relationships between conspecifics, resources, and HR use should show temporal variation associated with seasonal breeding (Gittleman and Thompson 1988).

Multiscale approaches have recently been used to study spatiotemporal effects of food abundance and abiotic factors on HR determination (Börger et al. 2006a; López‐Bao et al. 2010; van Beest et al. 2011; Campioni et al. 2013; Morellet et al. 2013; Campos et al. 2014; Godsall et al. 2014). However, few studies of free‐ranging animals have been able to simultaneously assess the effect of food and conspecific density on individual spacing behavior, as these two factors are often strongly correlated in natural systems (Benson et al. 2006). Furthermore, sex‐specific space use patterns are expected to emerge when the fitness of one sex is largely determined by resources for offspring provisioning, while the other is largely regulated by mating opportunities (Emlen and Oring 1977; Clutton‐Brock and Harvey 1978). Consequently, within‐species studies that simultaneously examine the sex‐specific effects of conspecific density and resource distribution on multiple spatiotemporal scales are largely lacking.

In this study, we assessed sex‐specific spatiotemporal influences of prey and conspecific density on variation in HR size for a solitary predator, the Eurasian lynx (*Lynx lynx*). We used individual location data from large‐scale and long‐term telemetry studies (1996–2012) in Sweden and Norway. Because of population expansion and active management of lynx (i.e., hunting) in this system, we had uncorrelated variation in prey density and conspecific density across male and female HRs, allowing us to investigate three main questions. First, do lynx show sex‐specific relationships in how prey and conspecific density influence total HR size? Females should reduce their HR size as prey and conspecific density increase (Adams 2001; Mitchell and Powell 2004), whereas males' HR size should mainly be affected by conspecific density as males' HR is expected to be set to maximize mating opportunities (Sandell 1989). Second, how do prey and conspecific densities influence sex‐specific HR sizes relative to the intensity of space use within the HR? For this, we compared five spatial scales of increasing intensity of HR use (Fig. 1A), with an expectation of contrasting patterns between the sexes as females' and males' HR size should be regulated by different factors at the total HR scale (Fig. 1B). Finally, we examined sex‐specific within‐year temporal effects on HRs. We compared mating and nonmating seasons to test whether males increase their HR size during the mating season (i.e., roaming; cf. Sandell 1989). For females, we expected no effect of mating season, but that the effect of prey density on HR size during suckling and kitten rearing would be stronger for reproducing compared to nonreproducing females.

## Materials and Methods

### Study system

Eurasian lynx in Scandinavia were almost hunted to extinction by the early 20th century, but due to legal protection and hunting restrictions, they have substantially recovered during the last decades and are now widespread throughout Sweden and Norway, with a total population estimate ~1800–2300 individuals in 2011 (Chapron et al. 2014). The lynx is a solitary and polygamous carnivore that displays intrasexual territoriality, although there may be some degrees of intrasexual HR overlap (Mattisson et al. 2011). Lynx mate in March (Mattisson et al. 2013) and give birth in late May/early June, and females give birth for the first time at the age of 2 (Nilsen et al. 2011). Juveniles become independent at 8–10 months, and most subadults have settled at 18 months of age (Samelius et al. 2011).

We used location data from 1998 to 2010 (Sweden) and 1996 to 2012 (Norway) from the south‐central part of the Scandinavian Peninsula (57°–63°N, 9°–17°E) for resident animals ≥20 months old. The study area encompasses a north–south environmental gradient where primary productivity, roe deer abundance (*Capreolus capreolus*: the primary prey for lynx in this region; Odden et al. 2006, 2013; Gervasi et al. 2014), proportion of agricultural land, and human density increase to the south, whereas the period with snow cover increases to the north. For a detailed description of the study areas, see Andrén et al. (2006) and Odden et al. (2013).

Lynx were captured and immobilized using strict ethics‐approved handling protocols (see Andrén et al. 2006; Arnemo et al. 2012). Animals were fitted with VHF transmitters (1996–2008: VHF collars MOD335 and MOD400NH), intraperitoneal transmitters (IMP/150/L and IMP/400/L; Telonics, Mesa, AZ, USA) or GPS collars (2003–2014; GPS plus mini, Vectronics Aerospace, Berlin, Germany; Lotek 3300SL; Lotek Wireless, Newmarket, Ontario, Canada; Televilt Posrec 300 and Tellus 1C, Followit, Lindesberg, Sweden).

Reproductive status for female lynx ≥2 years was checked annually using telemetry locations in May–June to find the natal lair. Kitten survival was determined by snow tracking in November–January (i.e., changes in litter size). For detailed description of determination of reproductive status and kitten survival, see Gaillard et al. (2014).

### Home range size estimation and spatiotemporal scale

We estimated lynx HR (km^2^) using the fixed‐kernel method (Worton 1989) with the “*adehabitatHR*” package (Calenge 2006) in R (R Core Team 2014). The kernel method estimates an utilization distribution (UD); consequently, kernel HR estimations are obtained as a function of an individual's relative use of space (Marzluff et al. 2004). From the UD, an animal's HR is defined as the smallest area that accounts for a specific proportion (isopleth) of the animal's total use of space; thus, an animal's intensity of use of the area increases with decreasing isopleth values (Fig. 1A). We estimated lynx total HR as the 90% isopleth using the reference bandwidth multiplied by 0.8 (Kie et al. 2010, 2013) to explore the influence of prey and conspecific density on annual (i.e., 1st February in year *t* to 31st January in year *t *+* *1) and seasonal basis. Furthermore, we calculated the 80%, 70%, 60%, and 50% isopleths to examine how the effect of prey density and conspecific density on area used changes with increasing intensity of space use within the HR (Fig. 1).

During the study period, the number of locations acquired per individual varied extensively as radiotracking technology developed. Due to the value of long‐term, individual‐based ecological studies (Pelton and van Manen 1996; Clutton‐Brock and Sheldon 2010), we included animals monitored with both GPS and VHF technology. To reduce biases from different sampling frequencies between animals and years (Börger et al. 2006b), we randomly sampled 1 location/day/individual. Mean (±SE) annual locations per individual were 83 ± 7.4. We only included animals with ≥25 locations and monitored ≥7 months (annual) or ≥half the season (seasonal), resulting in a total of 157 annual HRs for 77 individual lynx. For each individual with >100 annual locations, we randomly subsampled from 10 to 100 locations, resampled 200 times, to calculate the mean proportion of reference area (all annual locations/individual) included in HR size estimates in relation to number of locations used. Mean proportion of reference area (±SD) and mean coefficient of variation (±SD) for 25 locations were 0.85 ± 0.04 and 0.12 ± 0.03, compared to 0.97 ± 0.02 and 0.05 ± 0.02 for 83 locations. Although the number of locations per individual differed depending on collar technology (VHF = 64 ± 3, range: 25–175; GPS = 230 ± 12, range: 120–333), there was no effect of collar type on annual HR size (models including collar type compared to null models: ΔAIC_c_ = 3.4, *w*_*i*_ = 0.15).

We calculated mating (February 1 to April 15; males: *n* = 18; females: *n* = 22) and nonmating season HRs (April 16 to January 31; males: *n* = 28, females: *n* = 55). Although lynx mate in March, the annual and mating season HR calculations began in February to buffer potential premating behavioral changes just before mating (i.e., searching for or guarding mates). For females, we also calculated suckling (May 20 to September 30 representing birth to end of lactation, *n* = 71) and rearing seasonal HRs (May 20 to January 31 representing birth to independence: *n* = 44).

### Prey and conspecific density indices

We used reported yearly number of hunted roe deer (i.e., hunting bag) at the hunting district level in Sweden (Swedish Association for Hunting and Wildlife Management, available at: www.jagareforbundet.se) and municipality level in Norway (Statistics Norway, available at: www.ssb.no) as a proxy for prey density (Appendix S1). For conspecific density, we used lynx monitoring results where density of lynx family groups (i.e., female with kittens) is estimated at a regional scale based on snow tracking in January and February each year (Linnell et al. 2007). We calculated a HR‐specific annual prey density index as the area‐weighted average annual roe deer bag size across the hunting districts (Sweden) or municipalities (Norway) overlapping each annual HR (Sweden 13–123; Norway 0.5–81 shot roe deer per 10 km^2^). Similarly, we calculated the conspecific density index as the area‐weighted annual number of lynx family groups across the biogeographical regions (Sweden) or carnivore management areas (Norway) overlapping each HR (Sweden 0–4; Norway 0.23–0.5 family groups per 1000 km^2^). Because lynx monitoring focuses on family groups, there can be annual lynx HRs with zero lynx density (i.e., males and/or females without kittens; 4 home ranges of 157).

During the study period, the national population management goals were 300 and 65 family groups for Sweden and Norway, respectively, resulting in higher lynx hunting quotas and lower lynx density in Norway compared to Sweden (Ministry of the Environment 2003; Andrén et al. 2006; Linnell et al. 2010; SEPA 2013). The high hunting quotas in Norway in combination with the ongoing southward expansion of the Swedish lynx population (Samelius et al. 2011) resulted in uncorrelated prey and conspecific densities (compared to the null model: ΔAIC_c_ = 8.6, *w*_*i*_ = 0 for Norway and ΔAIC_c_ = 4.0, *w*_*i*_ = 0.12 for Sweden), allowing us to simultaneously study their effects on lynx HR size.

### Statistical analyses

**Figure 2:**
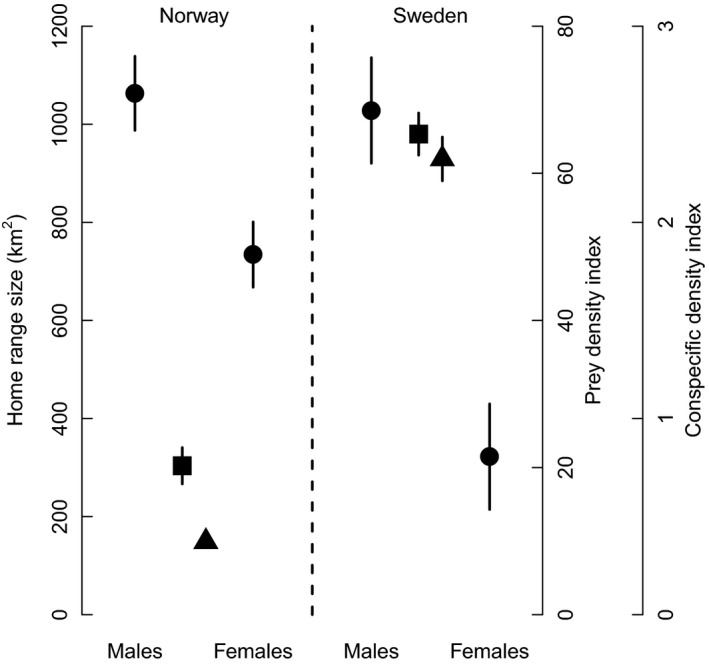
Country‐specific mean (±SE) prey density index (roe deer; squares), conspecific density index (triangles), and male and female lynx home range size (circles). Estimates are based on raw data.

We used general linear mixed models with a Gaussian error distribution using the “*lme4*” package (Bates et al. 2014) in R with log‐transformed HR size as the response variable. Individual identity and year were fitted as random effects in all models to account for repeated measurements. Log‐transformed prey and conspecific density indices were included as covariates together with their pairwise interactions with sex. Because of contrasting management regimes in Sweden and Norway (i.e., lynx population goals and hunting quotas), we also included country and the interaction between country and sex. Although prey and conspecific density varied between countries, there was no support for the interaction between country and prey or conspecific densities on annual HR size (Fig. 2; Table S1) so these interactions were not further considered. Furthermore, there was no support for additional latitudinal patterns in HR size not explained by prey or conspecific density (best model including latitude ΔAIC_c_ = 22.2; variable relative importance weight for latitude = 0, cf. Table 1).

**Table 1:** Highest ranked candidate models relating annual lynx home range (HR) size (*n* = 157) to conspecific density (lynx; L), prey density (roe deer; R), country (C), latitude (Lat), sex (S; difference of females from males), and interactions (*). The 90% kernel isopleth represents the total HR and decreasing isopleth values represents an increasing intensity of HR use (Fig. 1). For each model, we show sample‐size corrected AIC (AIC_c_), difference in AIC_c_ relative to the highest ranked model (ΔAIC_c_), and AIC weights (*w*_*i*_). For simplicity, only models with *w*_*i*_ > 0.01, univariate models, and intercept‐only models are shown

Spatial scale	90% isopleth, total HR			80% isopleth			70% isopleth			60% isopleth			50% isopleth		
Model	AIC_c_	ΔAIC_c_	*w*_*i*_	AIC_c_	ΔAIC_c_	*w*_*i*_	AIC_c_	ΔAIC_c_	*w*_*i*_	AIC_c_	ΔAIC_c_	*w*_*i*_	AIC_c_	ΔAIC_c_	*w*_*i*_
L* *+* *R* *+* *S* *+* *R*S	**216.8**	**0.0**	**0.37**	**225.2**	**0.0**	**0.3**	**235.6**	**0.0**	**0.24**	**247.1**	**0.5**	**0.19**	**257.9**	**0.9**	**0.15**
L* *+* *R* *+* *S	219.1	2.3	0.11	**226.2**	**1.0**	**0.18**	**235.7**	**0.1**	**0.22**	**246.6**	**0.0**	**0.24**	**257.0**	**0.0**	**0.24**
R* *+* *S* *+* *R*S	219.8	3.0	0.08	228.8	3.6	0.05	239.4	3.8	0.03	251.0	4.4	0.03	261.8	4.8	0.02
L* *+* *R* *+* *S* *+* *L*S* *+* *R*S	220.4	3.6	0.06	228.8	3.6	0.05	239.1	3.5	0.04	250.6	4.0	0.03	261.4	4.4	0.03
C* *+* *L* *+* *R* *+* *S* *+* *R*S	220.7	3.9	0.05	229.2	4.0	0.04	239.5	3.9	0.03	250.9	4.3	0.03	261.7	4.7	0.02
C* *+* *L* *+* *R* *+* *S* *+* *C*S	221.2	4.4	0.04	229.7	5.0	0.03	239.8	4.2	0.03	251.0	4.4	0.03	261.6	4.6	0.03
C* *+* *S* *+* *C*S	221.3	4.5	0.04	229.5	4.3	0.04	239.1	3.5	0.04	249.9	3.3	0.05	259.9	2.9	0.06
C* *+* *L* *+* *S* *+* *C*S	221.5	4.7	0.03	229.7	4.5	0.03	239.3	3.7	0.04	250.3	3.7	0.04	260.5	3.5	0.04
C* *+* *R* *+* *S* *+* *R*S	221.7	4.9	0.03	230.0	4.8	0.03	240.2	4.6	0.02	251.5	4.9	0.02	261.9	4.9	0.02
L* *+* *S	221.9	5.1	0.03	229.1	3.9	0.04	238.2	2.6	0.06	249.0	2.4	0.07	259.3	2.3	0.08
L* *+* *R* *+* *S* *+* *L*S	222.0	5.2	0.03	229.2	4.0	0.04	238.6	3.0	0.05	249.5	2.9	0.06	259.8	2.8	0.06
C* *+* *L* *+* *R* *+* *S	222.7	5.9	0.02	229.9	4.7	0.03	239.4	3.8	0.03	250.3	3.7	0.04	260.7	3.7	0.04
S	234.9	18.1	0.00	242.7	17.5	0.00	252.1	16.5	0.00	262.8	16.2	0.00	273.1	16.1	0.00
L	260.7	43.9	0.00	267.6	42.4	0.00	276.7	41.1	0.00	287.5	40.9	0.00	298.0	41.0	0.00
C	265.5	48.7	0.00	272.2	47.0	0.00	281.2	45.6	0.00	291.8	45.2	0.00	301.9	44.9	0.00
Intercept only	269.7	52.9	0.00	277.0	51.8	0.00	286.3	50.7	0.00	297.0	50.4	0.00	307.4	50.4	0.00
R	271.3	54.5	0.00	278.4	53.2	0.00	287.7	52.1	0.00	298.5	51.9	0.00	309.0	52.0	0.00
Lat	294.2	77.5	0.00	301.4	76.19	0.00	310.9	75.29	0.00	321.8	75.2	0.00	332.3	75.33	0.00

The models used for model average parameter estimates for each isopleth are indicated in boldface.

To test for seasonal HR size differences, we compared mating and nonmating seasons (males and females) and suckling and rearing seasons (females). For females, we initially included reproductive status as a three‐level explanatory factor (i.e., reproducing with surviving kittens; reproducing but lost all kittens; nonreproducing). However, there was no HR size differences between the two classes of reproducing females (models including kitten survival compared to null models: ΔAIC_c_ = 1.75, *w*_*i*_ = 0.29 for suckling season and ΔAIC_c_ = 4.55, *w*_*i*_ = 0.09 for rearing season); therefore, we included female reproductive status as a two‐level factor (reproducing and nonreproducing).

Candidate models were compared using the sample‐size corrected Akaike information criterion (AIC_c_) and AIC weights (*w*_*i*_) from the “*MuMIn*” package (Bartoń 2013) in R. Models with ΔAIC <2 were used to generate model‐averaged parameter estimates (Burnham and Anderson 2002). We used a bootstrap method implemented in R using the “*ez*” package (Lawrence 2013) to calculate 95% confidence intervals for mixed models. We used AIC weights on the full candidate model set to generate relative variable importance weights (RVI) for each explanatory variable. Model residuals did not violate assumptions for normality, homogeneity of variance, and structure relative to predictors. Means are presented with standard errors unless otherwise stated.

## Results

**Figure 3:**
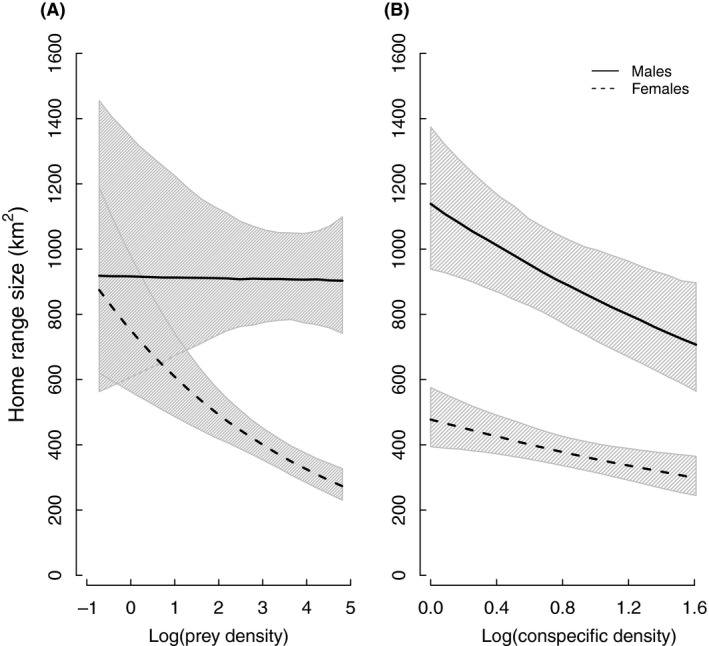
Sex‐specific relationships between annual lynx home range size (km^2^; 90% fixed‐kernel isopleth) and (A) prey density (i.e., roe deer), and (B) conspecific density. Model‐averaged predictions derived from the highest ranked models from Table 1 are shown (solid lines = males, dashed lines = females) with associated 95% CIs (see Table 2 for parameter estimates), where all other explanatory variables were held at their mean values. Home range size predictions were back‐transformed to their normal scale for the figure.

There were clear sex‐specific differences in annual HR size (90% isopleth males = 1045 ± 66 km^2^, range: 303–2290, *n* = 57; females = 483 ± 35 km^2^, range: 109–1853, *n* = 100), with range size dramatically decreasing with increased intensity of space use for both sexes (80%, 70%, 60%, and 50% isopleth area (km^2^): 748 ± 48, 566 ± 37, 432 ± 29, and 325 ± 22 for males and 343 ± 25, 255 ± 19, 192 ± 15, and 142 ± 11 for females). Total annual HR size for both males and females was negatively related to conspecific density (Fig. 3; Table 1). However, prey density affected female, but not male total HR size, with HR decreasing with increasing prey density (Fig. 3). Although female total HRs (90% isopleth) were larger in Norway compared to Sweden (Norway = 734 ± 67 km^2^, range; 225–1853, *n* = 39; Sweden = 322 ± 108, range: 109–733, *n* = 61), this difference was explained by conspecific and prey density, and not by country (RVI: prey = 0.86, conspecific = 0.81, country = 0.31; cf. Table 2).

**Table 2:** Relative variable importance (RVI) and model‐averaged parameter estimates with standard error (SE) for each variable retained in the best models for each HR isopleth in Table 1 (S = sex, R = prey density, L = conspecific density)

Parameter	90% isopleth, total HR			80% isopleth			70% isopleth			60% isopleth			50% isopleth		
	RVI	Estimate	SE	RVI	Estimate	SE	RVI	Estimate	SE	RVI	Estimate	SE	RVI	Estimate	SE
Intercept		7.04	0.21		6.91	0.28		6.68	0.27		6.45	0.27		6.20	0.26
S	1.00	−0.21	0.24	1.00	−0.51	0.36	1.00	−0.61	0.35	1.00	−0.67	0.34	1.00	−0.73	0.32
R	0.86	−0.00	0.06	0.83	−0.06	0.07	0.78	−0.07	0.07	0.74	−0.08	0.07	0.69	−0.09	0.07
L	0.81	−0.29	0.10	0.84	−0.33	0.11	0.85	−0.34	0.11	0.84	−0.36	0.11	0.82	−0.37	0.12
R*S	0.62	−0.20	0.07	0.5	−0.12	0.04	0.39	−0.09	0.04	0.32	−0.08	0.03	0.26	−0.06	0.03

### Sex‐specific intensity of space use effects

**Figure 4:**
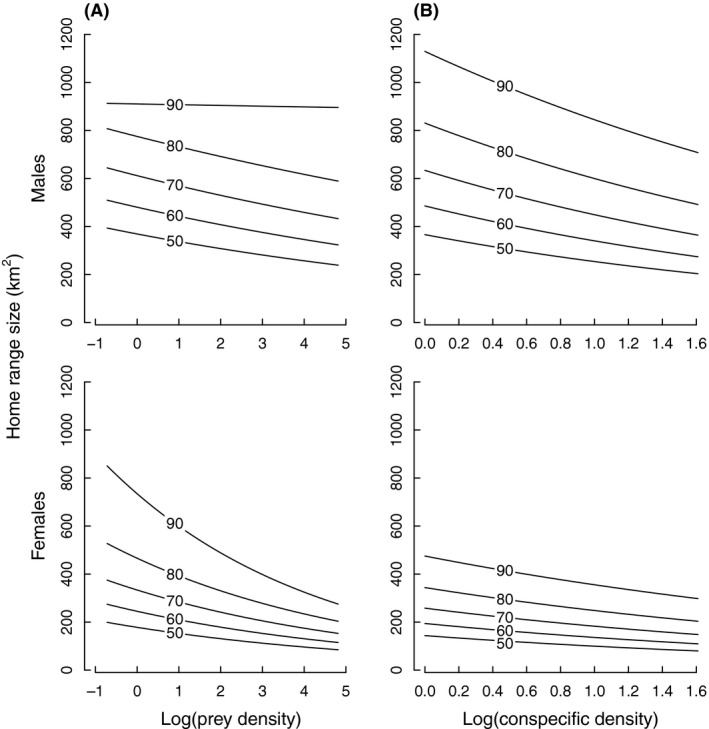
Sex‐specific relationships between annual home range (HR) size (km^2^) and (A) prey density (i.e., roe deer), and (B) conspecific density for a range of isopleths (90, 80, 70, 60, and 50%) that represent increasing intensity of use of the HR (Fig. 1). The lines show model‐averaged predictions for the different isopleth levels from Table 1, with all other explanatory variables kept at their mean values. HR size predictions were back‐transformed to their normal scale for the figure. For model parameters, see Table 2.

As predicted, both prey and conspecific density showed spatial scale‐dependent effects on HR size, with the largest difference in sex‐specific effect of prey density on HR size at the 90% isopleth (Fig. 4; Table 1). For females, the negative effect of prey on range size decreased with increasing intensity of space use, while males showed the opposite pattern with the negative effect of prey density on range size becoming evident for high intensity of space use (Fig. 4). For males, the proportion of the total HR encompassed by the highest intensity of space use (50% and 60% isopleths) decreased with increasing prey density, but this effect was not found for other isopleth area ratios (Fig. S1; Table S2). The negative relationship between conspecific density and HR size was evident for both sexes, but this effect did not decrease with increasing intensity of space use (Fig. 4; Table 1).

### Seasonal effects

Contrary to our expectations, males' HR size was smaller during the mating compared to nonmating season (789 ± 68 vs. 1029 ± 93 km^2^), while females' HR size was larger during the mating season (647 ± 112 vs. 486 ± 53 km^2^; Table 3). Reproducing females had smaller HRs compared to nonreproducing females during both suckling and rearing periods (Table S3). Prey density was not related to HR size during suckling, but it was negatively related to the rearing season HR size. Conspecific density was negatively related to female seasonal HR size, regardless of reproductive status (Table S3).

**Table 3:** Full candidate models testing the influence of sex (S; difference of females from males) and season (M; difference of mating season from nonmating season) on lynx seasonal home range (HR) size. Seasonal HRs are estimated as the 90% fixed‐kernel isopleth. Terms are as in Table 1

Model	AIC_c_	ΔAIC_c_	*w*_*i*_
S* *+* *M* *+* *S*M	1784	0.0	1.00
S* *+* *M	1798	14	0.00
S	1807	23	0.00
M	1824	40	0.00
Intercept only	1833	49	0.00

Model parameter estimate (±SE) for highest ranked model: Seasonal home range size = 810 ± 101 − 558 ± 12 *S − 217 ± 100 *M + 282 ± 133 * S*M.

## Discussion

By simultaneously examining prey and conspecific density in a spatiotemporal context, we show that new insights can be found in the study of sex differences in spacing behavior. The importance of being able to account for both prey and conspecific density when studying HR size should not be underestimated, as this allowed us to demonstrate that observed differences in total HR size between Sweden and Norway (Fig. 2) were completely explained by different prey and conspecific densities. Furthermore, we show that the effect of prey density on total HR size is restricted to females, in contrast to a previous study that did not account for the confounding effects of conspecific density and found a negative relationship between roe deer density and total HR size for both male and female lynx (Herfindal et al. 2005). By assessing sex‐specific range size determinants as intensity of space use increased within the HR, we could show that it is only at higher isopleth levels (50–60%) that male space use is influenced by prey density.

Females' total HR size decreased as prey density increased, supporting the expectation that females adapt their space use relative to the resources needed to survive and successfully reproduce (Sandell 1989). However, the influence of prey density on area use decreased as intensity of space use increased within the HR (Fig. 4). This indicates that although food availability is a key driver of total HR size for females, factors other than food define female space use in the more intensively used areas (e.g., availability of den sites, or habitats that provide protection for females and their offspring from human intrusion and intraguild predation; Kelt and Van Vuren 2001; Basille et al. 2013; Rauset et al. 2013). Because areas that provide protection and den sites are commonly in steep, rugged terrain or dense forest (Rauset et al. 2013), they may represent local habitats with little variation in prey density. Thus, although intensively used areas are often assumed to contain high and predictable prey densities (e.g., Maher and Lott 2000; Powell 2000), our results show that this is not necessarily the case because it was the size of the outer area of the females' HR that responded strongest to changes in prey density (Fig. 4; Table 2). This suggests that it is the less intensively used areas (i.e., those relating to the total HR size) that are critical for food provisioning. The fact that lynx select different habitats to rest during the day or between kills compared to hunting (Bouyer et al. 2015) could explain this decoupling of intensively used areas from prey density.

For males, that prey density did not affect total HR size supports the expectation that male large‐scale space use is primarily driven by access to mates, not food (Sandell 1989). However, a negative relationship between prey density and male range size became visible with increasing intensity of space use due to energetic requirements (Fig. 4). This is also supported by the proportion of the total HR included in the 50% isopleth area being negatively correlated with prey density for males but not females (Fig. S1; Table S2). Furthermore, when males' area use was similar to females' total HR size (i.e., males' 60% isopleth = 432 ± 29 vs. females' total HR size = 483 ± 35 km^2^), the interaction between prey density and sex was not included in the best model (Table 1).

Our results show scale‐dependent, sex‐specific effects of different resources on spacing behavior, corresponding to the scale‐dependent habitat selection suggested by Rettie and Messier (2000) to reflect the hierarchy of fitness‐limiting factors. At a finer spatial scale (within HR), the importance of different space use determinants will be conditional on the coarser scale (total HR) to maximize an individual fitness (i.e., for females' total HR = food requirements, 50% isopleth = shelter/protection; for males' total HR = access to females, 50–60% isopleth = food requirements).

Contrary to our predictions, the effect of conspecific density did not change with intensity of space use for either sex (Fig. 4; Table 2) suggesting that intrasexual interactions may occur in the area between the 50% isopleth and the HR borders. For females, the negative effect of conspecific density on HR size likely results from intrasexual competition (Maher and Lott 2000; Benson et al. 2006). For males, however, the relationship between HR size and conspecific density is probably driven by two factors: that is, reduced maximum HR size as conspecific density increases due to the cost of increased competition and increasing HR size at low conspecific density to increase their encounters with females. Total HR size of male lynx did not adapt to encompass a similar number of female HRs as conspecific density changed (Fig. 2), contrary to bobcats (*Lynx rufus*) that exhibit an isometric relationship between male and female HRs (Ferguson et al. 2009). Instead, the ratio between male and females' HR size was positively related to prey density in our study. Consequently, male lynx in areas with high prey density encounter more females compared to males in low prey density areas where males and females HRs are more similar in size. This suggests that male lynx have an upper bound for their HR size, likely due to the energetic costs of maintaining large territories and increased risk of mortality associated with using unfamiliar areas that outweighs any additional fitness benefits of encountering more females (Kelt and Van Vuren 2001).

We found that males' HR during the mating season was smaller than during nonmating season, indicating that male lynx do not generally adopt a roaming mating tactic. We suggest that this behavioral pattern is because female Eurasian lynx [as well as Canadian lynx (*L. canadensis*) and Iberian lynx (*L. pardinus*)], contrary to other felids, are strictly seasonal breeders due to a mono‐estrous cycle (Jewgenow et al. 2014; Painer et al. 2014). Hence, males move over smaller areas and interact more when they stay close to receptive females during a short mating season, whereas they keep larger exclusive HRs during the rest of the year to reduce the presence of competing males before the mating season. This is also supported by (1) observations of lethal male‐male interactions during the mating season (Mattisson et al. 2013), (2) that male lynx only show moderate seasonal changes in hormonal levels related to reproductive capacity (Müller et al. 2014), and (3) that male total annual HR size is negatively affected by conspecific density but not by prey density.

Because the most energy‐consuming activities for females are lactation and feeding young (Gittleman and Thompson 1988), there is an expectation that prey density effects on HR size should be strongest during these critical periods of high energetic requirements (Sandell 1989). However, females with kittens had consistently smaller seasonal HRs than nonreproducing females, and the effect of prey density on HR size was similar for reproducing and nonreproducing females at both seasonal and annual time scales. Thus, reductions in HR size for reproducing females during suckling is likely due to young, dependent kittens limiting the mother's mobility (Dahle and Swenson 2003) as well as female lynx avoiding human disturbance during this period (White et al. 2015). That nonreproducing females did not reduce their HR size during summer despite an increase in prey availability (i.e., small prey and domestic sheep; Odden et al. 2006, 2013; Gervasi et al. 2014) suggests that nonreproducing female HR size is regulated by prey availability during the winter.

Our results highlight the importance of simultaneously considering resources and intraspecific interactions as determinants of animal spacing patterns. By examining variation in intensity of space use, instead of only focusing on total HR and/or an arbitrarily chosen core area (usually 50‐ or 30% isopleth for kernel HR estimations; Vander Wal and Rodgers 2012), we show that large knowledge gains are still to be made in the study of spacing behavior. We recommend a spatiotemporal approach be used in future HR studies, as it highlights how the use of different resources varies in importance within an animal's HR. Consequently, factors that may not be related to total HR size still may be important determinants in animal spatial ecology. In turn, this will lead to better models of ecological systems to both inform theory and management.

## Conflict of Interest

None declared.

## Data Accessibility

Relevant data for this study will be archived in the Dryad Digital Repository conditional on acceptance.

## Supporting information


**Appendix S1.** The use of yearly roe deer hunting bags as proxy for roe deer density.
**Figure S1.** Proportion of 90% isopleth area included in the 50% isopleth for male in relation to prey density index.
**Table S1.** Model selection relating lynx annual home range size to (a) the interaction between country and prey density index and (b) the interaction between country and conspecific density index.
**Table S2.** Model selection relating sex‐specific annual home range isopleth area‐ratios to prey density index and country.
**Table S3.** Model selection relating lynx female seasonal home range size to reproductive status, prey density, conspecific density and country.
