# Supplementary material for: Intensity of space use reveals conditional sex‐specific effects of prey and conspecific density on home range size
Source: Ecol Evol. 2016 Mar 28;6(9):2957–67. doi: 10.1002/ece3.2032 (PMC4863019; doi:10.1002/ece3.2032)
Supplement: Supplementary file 1 — Appendix S1. The use of yearly roe deer hunting bags as proxy for roe deer density. Figure S1. Proportion of 90% isopleth area included in the 50% isopleth for male in relation to prey density index. Table S1. Model selection relating lynx annual home range size to (a) the interaction between country and prey density index and (b) the interaction between country and conspecific density index. Table S2. Model selection relating sex‐specific annual home range isopleth area‐ratios to prey density index and country. Table S3. Model selection relating lynx female seasonal home range size to reproductive status, prey density, conspecific density and country. [file ECE3-6-2957-s001.docx]

**Supporting information**

**Appendix 1**

Aronsson, M., Low, M., López-Bao, J.V., Persson, J., Odden, J., Linnell, J.D.C & Andrén, H.

Intensity of space use reveals conditional sex-specific effects of prey and conspecific density on home range size

Roe deer hunting bags statistics are a good functional proxy for prey density in across the large areas (lynx HR) used in this study for the following reasons: (i) roe deer harvest bag size is significantly correlated with roe deer pellet group counts in both Sweden (pellet groups counts carried out every second year from 1996 to 2008 and harvest bag size; Pearson correlation coefficient, r = 0.85, df=42, p < 0.0001) and Norway (Bouyer *et al.* 2015). (ii) There is a high concordance between hunting bag size and three other independent indexes of roe deer abundance (i.e. estimated population size, traffic-killed roe deer, sightings at feeding stations; Grøtan *et al.* 2005). (iii) In Sweden, since the 1980s there is an open hunting season with no hunting bag limits for roe deer, with local hunting bag records collected using the same reporting system over time, ensuring consistency between years and areas. (iv) In Norway the roe deer harvest is regulated through a quota system, but only a low fraction of the annual quotas are actually harvested, thus hunting bag size will be more influenced by roe deer density rather than quotas per se (Grøten et al. 2005). (v) Melis et al. (2013) found that roe deer harvest rates was not affected by roe deer density and lastly (vi), yearly hunting bag statistics obtained on a hunting district/municipality level can be generalized to deer density across large areas such as lynx HR (hundreds of km^2^; see results) since both hunting districts and municipalities within the study area are considerably smaller than lynx HR sizes, resulting in multiple hunting districts/municipalities within each lynx HR (Fig. A1). Hence, the use of area-weighted average annual roe deer bag size across the hunting districts/municipalities overlapping each annual lynx HR

Bouyer, Y. et al. 2015. Using Zero-Inflated Models to Predict the Relative Distribution and Abundance of Roe Deer Over Very Large Spatial Scales. – Ann. Zool. Fenn. 52:66–76.

Grøten, V. et al. 2005. Climate causes large-scale spatial synchrony in population

fluctuations of a temperate herbivore. – Ecology 86:1472–1482.

Melis C, Nilsen EB, Panzacchi M, Linnell JDC, Odden J (2013) Roe deer face competing risks between predators and along a gradient in abundance. Ecosphere 4: e111. Doi: 10.1890/ES13-00099.1


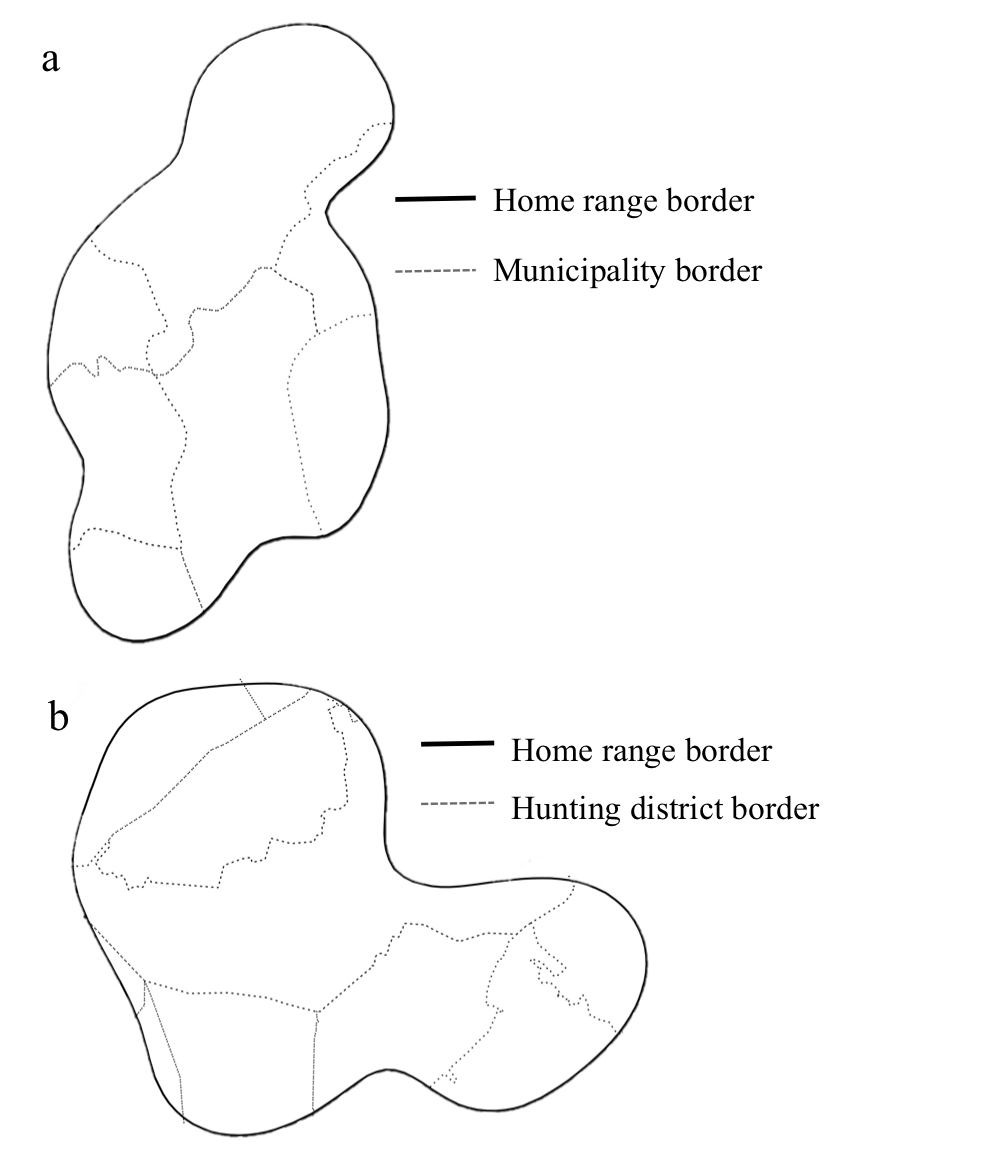


**Figure A1.** Lynx home ranges overlap multiple municipalities (Norway, a) or hunting districts (Sweden, b).

**Supporting information**

**Figures and tables**

Aronsson, M., Low, M., López-Bao, J.V., Persson, J., Odden, J., Linnell, J.D.C & Andrén, H.

Intensity of space use reveals conditional sex-specific effects of prey and conspecific density on home range size

**Figure S1.** Relationship between proportion of the 90% Isopleth area included in the 50% isopleth and prey density index. Line shows model prediction from highest ranked model in Table S4. Points show the raw data for Norway (○) and Sweden (●).

**Table S1.** Full candidate model set relating lynx annual home range size to (a) prey density (roe deer; R), country (C), and their interaction (*) and (b) conspecific density index (lynx; L), country, and their interaction Models are ranked based on sample-size corrected AIC (AIC_c_), difference in AIC_c_ relative to the highest-ranked model (ΔAIC_c_) and AIC-weights (*w_i_*).

| Model | AIC_c_ | ΔAIC_c_ | *w_i_* |
| --- | --- | --- | --- |
| (a) |  |  |  |
| C | 265.5 | 0.0 | 0.79 |
| Null | 269.7 | 4.2 | 0.10 |
| C+R | 271.0 | 5.5 | 0.05 |
| R | 271.3 | 5.8 | 0.04 |
| C+R+C*R | 272.6 | 7.1 | 0.02 |
| (b) |  |  |  |
| L | 260.7 | 0.0 | 0.65 |
| C+L+C*L | 263.6 | 2.9 | 0.15 |
| C+L | 263.8 | 3.1 | 0.14 |
| C | 265.5 | 4.8 | 0.06 |
| Null | 269.7 | 9.0 | 0.01 |

**Table S2.** Full candidate models relating sex-specific annual home range isopleth area-ratio (see text for details) to prey density (roe deer; R) and country (C). All models were fitted using Binomial error distributions and logit link function. Terms are as in table S1.

| Spatial scale | 50-90% isopleth area-ratio | | | 60-90% isopleth area-ratio | | | 70-90% isopleth area-ratio | | | 80-90% isopleth area-ratio | | |
| --- | --- | --- | --- | --- | --- | --- | --- | --- | --- | --- | --- | --- |
| Model | AIC_c_ | ΔAIC_c_ | *w_i_* | AIC_c_ | ΔAIC_c_ | *w_i_* | AIC_c_ | ΔAIC_c_ | *w_i_* | AIC_c_ | ΔAIC_c_ | *w_i_* |
| MALES |  |  |  |  |  |  |  |  |  |  |  |  |
| R | -181.73 | 0 | 0.44 | -169 | 0 | 0.35 | -166.88 | 1.02 | 0.23 | -194.75 | 1.62 | 0.18 |
| C+R | -180.89 | 0.84 | 0.29 | -168.69 | 0.31 | 0.30 | -167.50 | 0.40 | 0.31 | -195.94 | 0.44 | 0.32 |
| C | -180.02 | 1.71 | 0.19 | -168.43 | 0.57 | 0.26 | -167.89 | 0 | 0.37 | -196.37 | 0 | 0.40 |
| C+R+C*R | -178.29 | 3.45 | 0.08 | -166.11 | 2.89 | 0.08 | -164.92 | 2.98 | 0.08 | -193.34 | 3.04 | 0.09 |
| FEMALES |  |  |  |  |  |  |  |  |  |  |  |  |
| Intercept only | -290.44 | 0 | 0.45 | -273.58 | 0 | 0.50 | -277.95 | 0 | 0.54 | -333.11 | 0 | 0.55 |
| C | -288.68 | 1.76 | 0.19 | -271.63 | 1.95 | 0.19 | -275.88 | 2.07 | 0.19 | -330.94 | 2.17 | 0.19 |
| R | -288.44 | 2.00 | 0.16 | -271.47 | 2.11 | 0.18 | -275.74 | 2.21 | 0.18 | -330.92 | 2.19 | 0.18 |
| C+R | -288.30 | 2.13 | 0.15 | -270.37 | 3.22 | 0.10 | -273.91 | 4.04 | 0.07 | -328.67 | 4.43 | 0.06 |
| C+R+C*R | -286.05 | 4.38 | 0.05 | -268.05 | 5.5 | 0.03 | -271.80 | 6.15 | 0.02 | -326.87 | 6.23 | 0.02 |

**Table S3.** Highest-ranked candidate models relating female seasonal home range size (suckling season n=71; rearing season n=44) to reproductive status (Repr; where the estimate is the difference of reproducing from non-reproducing), conspecific density (lynx; L), prey density (roe deer; R), country (C; difference of Sweden from Norway), and interactions (*). Seasonal home ranges are estimated as the 90% fixed kernel isopleth. Terms are as in table S1, only models with AIC weights > 0.01, univariate models and intercept-only models are shown.

| Model | AIC_c_ | | ΔAIC_c_ | | *w_i_* | |  |
| --- | --- | --- | --- | --- | --- | --- | --- |
| SUCKLING SEASON | |  | |  | |  | |
| C+Repr | | 83.3 | | 0.00 | | 0.34 | |
| L+Repr | | 84.5 | | 1.20 | | 0.19 | |
| C+L+Repr | | 86.17 | | 2.87 | | 0.08 | |
| C+Repr+C*Repr | | 86.23 | | 2.93 | | 0.08 | |
| L+Repr+L*Repr | | 86.28 | | 2.98 | | 0.08 | |
| R+Repr | | 86.67 | | 3.37 | | 0.06 | |
| L+R+Repr | | 87.92 | | 4.62 | | 0.03 | |
| C+R+ Repr | | 88.22 | | 4.92 | | 0.03 | |
| C+L+Repr+L*Repr | | 88.35 | | 5.05 | | 0.03 | |
| Repr | | 88.98 | | 5.68 | | 0.02 | |
| C+L+Repr+C*Repr | | 89.24 | | 5.95 | | 0.02 | |
| L | | 108.26 | | 24.96 | | 0.00 | |
| C | | 111.84 | | 28.55 | | 0.00 | |
| R | | 112.41 | | 29.12 | | 0.00 | |
| Intercept only | | 114.30 | | 31.00 | | 0.00 | |
| REARING SEASON | |  | |  | |  | |
| L+R+Repr | | 104.32 | | 0.00 | | 0.31 | |
| L+Repr | | 106.22 | | 1.91 | | 0.12 | |
| C+Repr | | 106.41 | | 2.09 | | 0.11 | |
| C+L+Repr | | 107.09 | | 2.77 | | 0.08 | |
| C+R+Repr | | 107.31 | | 2.99 | | 0.07 | |
| C+L+Repr+Repr | | 107.63 | | 3.31 | | 0.06 | |
| L+R+Repr+L*Repr | | 107.69 | | 3.37 | | 0.06 | |
| R+Repr | | 108.49 | | 4.17 | | 0.04 | |
| C+Repr+C*Repr | | 109.46 | | 5.14 | | 0.02 | |
| L+Repr+L*Repr | | 109.55 | | 5.23 | | 0.02 | |
| C+L+Repr+C*Repr | | 110.03 | | 5.71 | | 0.02 | |
| L+R+Repr+R*Repr | | 110.05 | | 5.73 | | 0.02 | |
| L | | 114.22 | | 9.90 | | 0.00 | |
| C | | 116.82 | | 12.50 | | 0.00 | |
| R | | 119.49 | | 15.17 | | 0.00 | |
| Repr | | 120.58 | | 16.26 | | 0.00 | |
| Intercept only | | 128.03 | | 23.71 | | 0.00 | |

Model average parameter estimates ±SE for models with ΔAIC_c_ ≤2

Suckling: Log(home range size) = 6.6±0.18 – 1.21±0.18 R – 0.38±0.11 C – 0.20±0.064 LD.

Rearing: Log(home range size) = 6.9±0.23 – 0.48±0.12 R – 0.53±0.19 LD – 0.12±0.037 RDD
